# Magnetoelectricity in CoFe_2_O_4_ nanocrystal-P(VDF-HFP) thin films

**DOI:** 10.1186/1556-276X-8-374

**Published:** 2013-09-03

**Authors:** Xiaohua Liu, Shuangyi Liu, Myung-Geun Han, Lukas Zhao, Haiming Deng, Jackie Li, Yimei Zhu, Lia Krusin-Elbaum, Stephen O’Brien

**Affiliations:** 1 Department of Chemistry, The City College of New York, Marshak-1326, 160 Convent Ave, New York, NY 10031, USA; 2 Energy Institute, The City University of New York, New York, NY 10031, USA; 3 Department of Chemistry, The Graduate Center of CUNY, New York, NY 10016, USA; 4 Department of Mechanical Engineering, The City College of New York, Marshak-1326, 160 Convent Ave, New York, NY 10031, USA; 5 Department of Material Science and Condensed Matter Physics, Brookhaven National Laboratory, Building 480, Upton, NY 11973, USA; 6 Department of Physics, The City College of New York, 160 Convent Ave, New York, NY 10031, USA

**Keywords:** Thin film, Magnetoelectric nanocomposite, Magnetostrictive, P(VDF-HFP), CoFe_2_O_4_

## Abstract

Transition metal ferrites such as CoFe_2_O_4_, possessing a large magnetostriction coefficient and high Curie temperature (*T*_c_ > 600 K), are excellent candidates for creating magnetic order at the nanoscale and provide a pathway to the fabrication of uniform particle-matrix films with optimized potential for magnetoelectric coupling. Here, a series of 0–3 type nanocomposite thin films composed of ferrimagnetic cobalt ferrite nanocrystals (8 to 18 nm) and a ferroelectric/piezoelectric polymer poly(vinylidene fluoride-co-hexafluoropropene), P(VDF-HFP), were prepared by multiple spin coating and cast coating over a thickness range of 200 nm to 1.6 μm. We describe the synthesis and structural characterization of the nanocrystals and composite films by XRD, TEM, HRTEM, STEM, and SEM, as well as dielectric and magnetic properties, in order to identify evidence of cooperative interactions between the two phases. The CoFe_2_O_4_ polymer nanocomposite thin films exhibit composition-dependent effective permittivity, loss tangent, and specific saturation magnetization (*M*_s_). An enhancement of the effective permittivity and saturation magnetization of the CoFe_2_O_4_-P(VDF-HFP) films was observed and directly compared with CoFe_2_O_4_-polyvinylpyrrolidone, a non-ferroelectric polymer-based nanocomposite prepared by the same method. The comparison provided evidence for the observation of a magnetoelectric effect in the case of CoFe_2_O_4_-P(VDF-HFP), attributed to a magnetostrictive/piezoelectric interaction. An enhancement of *M*_s_ up to +20.7% was observed at room temperature in the case of the 10 wt.% CoFe_2_O_4_-P(VDF-HFP) sample.

## Background

Magnetoelectric materials, possessing spontaneous electric and magnetic ordering, show applications in multiple-state memory elements, magnetic field sensors, phase shifters, and microwave frequency transducers. Single-phase multiferroics, such as BiFeO_3_[1], YMnO_3_[2], and CdCr_2_S_4_[3], exhibit intrinsic magnetoelectric (ME) effect with inherent cross-coupling between magnetic and electric orders. However, such materials are empirically rare [4] and magnetoelectrically weak due to the contraindication between ferroelectricity and magnetism [5]. In addition, the observed ME effect is far below room temperature [6], which severely limits practical use in device fabrication. An alternative approach is two-phase composite films, in which the ferri/ferromagnetic and ferro/piezoelectric phases are combined. With various connectivity schemes (e.g., 0–3 particulate type, 2–2 laminate type, and 1–3 fiber/rod type), these heterostructures have offered the opportunity to tune ferroelectric and magnetic properties independently, and the ME coefficient is 3 orders of magnitude higher than their single-phase counterparts [7].

The magnetoelectric effect in most multiferroic composites is known as strain-mediated, in which the ME coupling is a concerted result of the piezoelectric effect from the piezoelectric phase and magnetostrictive effect from the magnetic phase. An electric field induces a distortion of the piezoelectric phase, which in turn distorts the magnetostrictive phase, generating a magnetic field and vice versa. Substantial ME coupling requires the ferroelectric phase to be in possession of a high piezoelectric coefficient, while the magnetic phase possess both high magnetostriction and resistivity, with an intimate mechanical contact between the two [8]. Ceramic composites have a combination of ferroelectric and magnetic oxides; polymer composites have the magnetic oxides embedded in ferroelectric polymer matrix. The former is limited by high dielectric loss resulting from the interface; the latter offers mechanical flexibility with facile processing. For instance, with high strength and good stability [9], polyvinylidene difluoride (PVDF) and its copolymers such as poly(vinylidenefluoride-co-trifluoroethylene) (P(VDF-TrFE)) [10] and poly(vinylidene fluoride-hexafluoropropylene) (P(VDF-HFP)) [11-13] are well known for their ferroelectricity and piezoelectricity, which make them ideal candidates for multiferroic film fabrication and ME effect exploration. Transition metal ferrites such as CoFe_2_O_4_, possessing a large magnetostriction coefficient (*λ* ≈ 10^−4^) [14] and high Curie temperature (*T*_c_ > 600 K) [15], serve as excellent candidates for the magnetic phase.

Although the mechanism of the magnetoelectric coupling is straightforward, complications arise when quantifying the details of polymer-based nanocomposites. The presence of polymorphism (e.g., *α*, *β*, *γ*, *δ* phases in PVDF), domain walls, grain boundaries, residual stain/magnetization, surface charge, and voids can significantly hinder the ME effect. Andrew and Clarke [16] found that the inclusion of well-dispersed Ni_0.5_Zn_0.5_Fe_2_O_4_ nanoparticles in a PVDF matrix can enhance the ferroelectric phase content. Liu et al. [17]. reported epitaxial BaTiO_3_-CoFe_2_O_4_ nanocomposite thin films (thickness, 100 nm) with phase transition mediated by tensile strain. Recently, a magnetoelectric coupling coefficient of 12 V/cm · Oe was obtained for P(VDF-HFP)/Metglas laminates [18]. Martins et al. [19] fabricated ferrites/PVDF nanocomposites films with thickness of 40 to 50 μm by solvent casting and melt processing. Guo and co-workers prepared particulate Ni_0.5_Zn_0.5_Fe_2_O_4_/P(VDF-TrFE) films (thickness, 50 to 60 μm) by wet chemistry routes, with observable magnetodielectric coefficients represented as a percentage change of dielectric constant with and without an external magnetic field [20].

In this work, we report a novel approach to fabricate 0–3 type particulate nanocomposite thin films composed of dispersed CoFe_2_O_4_ (CFO) nanoparticles embedded in P(VDF-HFP) matrix. Prepared through spin/cast-coating techniques, such films exhibit homogenous thickness ranging from 200 nm to 1.6 μm. With a focus on the potential for magnetoelectric coupling, the morphology, microstructure, dielectric, magnetic, and magnetoelectric properties are investigated systematically.

## Methods

The CoFe_2_O_4_ nanocrystals were synthesized by a hydrothermal route [21]. In a typical reaction, 2 mmol Co(NO_3_)_2_ · 6H_2_O (Aldrich, 98+%) and 4 mmol Fe(NO_3_)_3_ · 9H_2_O (Aldrich, 98+%) were first dissolved in deionized water. Ethanolamine was dropwise added in the solution until precipitation completed. The obtained precipitate was collected by centrifugation and washed with deionized water. Ammonium hydroxide was then added to re-dissolve the solids. The reaction mixture was transferred into a stainless steel autoclave, with 80% volume filled with the ammonium hydroxide solution. The autoclave was then heated at 200°C for 10 to 30 h. The resultant CoFe_2_O_4_ nanopowders were washed, collected, and dried in air at 60°C overnight.

The CoFe_2_O_4_/polymer nanostructured films were prepared via multiple spin coating and cast coating followed by thermal treatment. N,N-dimethylformamide was first used to dissolve CoFe_2_O_4_ nanoparticles and P(VDF-HFP) pallets or polyvinylpyrrolidone (PVP) powder separately, with concentration of 20 mg/ml. Then, the two suspensions were mixed under ultrasonification, according to the weight ratio of CFO versus polymer, and spin-coated or cast-coated on Si or glass substrates and dried at 90°C under vacuum. The thickness of the obtained thin films (200 nm to 1.6 μm) was controlled by the times and/or rotation speed (300 to 1000 rpm) of the spin coating. To measure film thickness, scanning electron microscopy (SEM) cross-sectional analysis was applied. The Si substrate was scored and cut/fractured in order to observe film cross sections, which were then easily analyzed by SEM. Correct instrumental calibration and review of the film over several regions confirmed thin film uniformity, expected for spin/cast coating, and thicknesses could be determined to within ±7%. For dielectric measurements, the glass substrates were pre-deposited with rectangular (1 mm × 5 mm) Ag bottom electrodes by a thermal evaporator. Top electrodes were deposited (5 mm × 1 mm) after the films were coated and dried, leaving the composite sandwiched between two electrodes with square crossed area of 1 mm × 1 mm.

The phase purity and crystal structure of the CoFe_2_O_4_ particles was analyzed by X-ray diffraction (XRD) with a PANalytical powder X-ray diffractometer (Almelo, The Netherlands) with Ni-filtered Cu K*α* radiation (*λ* = 1.54056 Å). The data were collected in step scanning mode from 20° to 80°. Microstructural characterization of the CFO powders was performed by transmission electron microscopy (TEM) with a JEOL 3000 F (Akishima-shi, Japan) with an accelerating voltage of 300 kV. We used a JEOL ARM 200CF equipped with cold field emission gun and spherical aberration correctors for both scanning transmission electron microscopy (STEM) and high-resolution transmission electron microscopy (HRTEM). Surface morphology, nanoparticle distribution, and film thickness of the CFO/polymer composite were evaluated by a Zeiss Supra 55VP SEM (Oberkochen, Germany). Dielectric measurements including frequency dependence of *ϵ*′, dielectric constant and tan *δ*, and dielectric loss were measured by an Agilent 4294A precision impedance analyzer. Magnetic measurements including zero field-cooled and field-cooled (ZFC/FC) low field magnetization versus temperature and room temperature hysteresis loops were carried out using a Quantum Design MPMS XL-5 SQUID magnetometer (San Diego, CA, USA), with applied fields up to 5 T and temperatures from 1.84 to 400 K.

## Results and discussion

**Figure 1:**
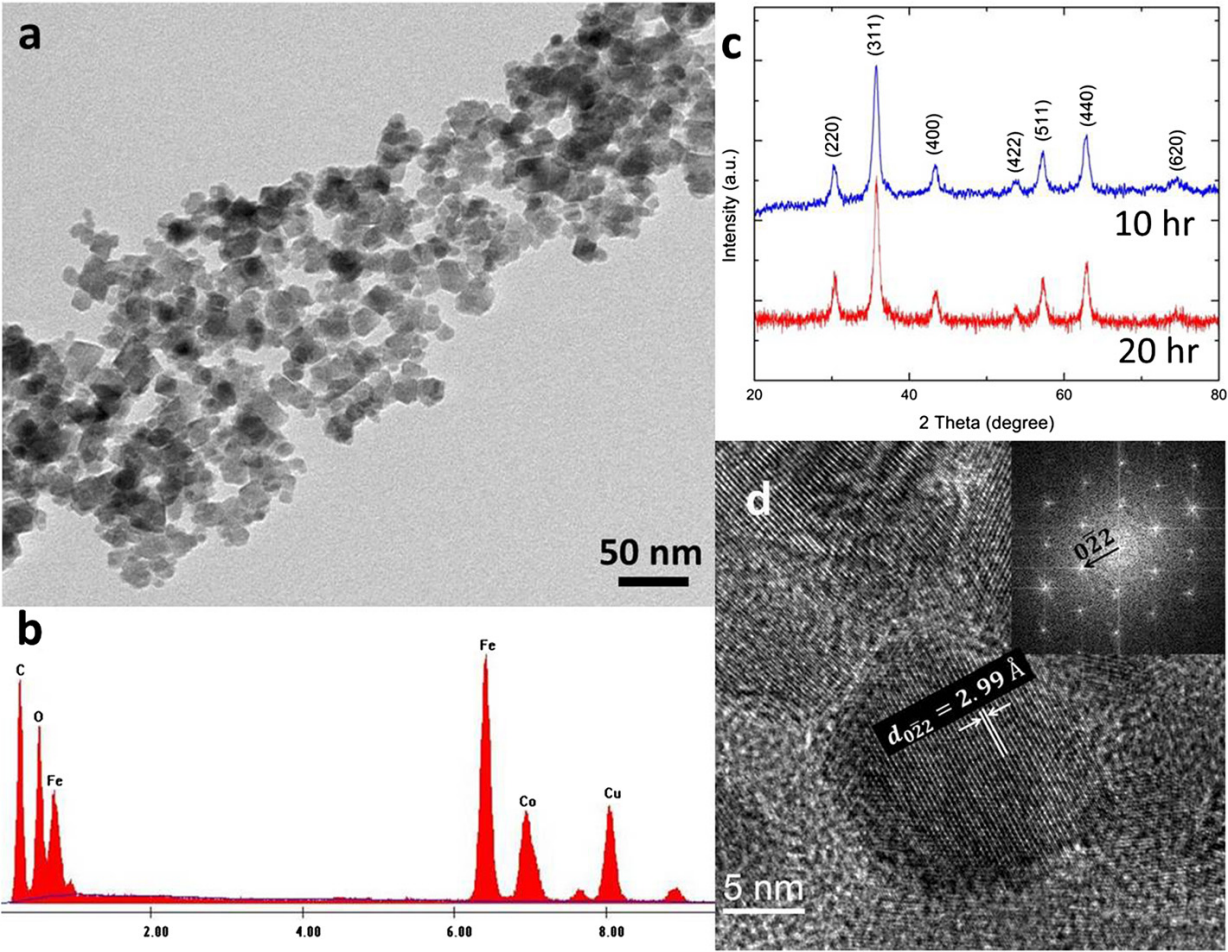
**TEM image**, **EDX spectra**, **XRD pattern**, **and HRTEM of CoFe**_**2**_**O**_**4 **_**nanocrystals.** Low magnification TEM image **(a)** of CoFe_2_O_4_ nanocrystals synthesized via a solvothermal process and its corresponding EDX spectra **(b)**. **(c)** XRD patterns of the CoFe_2_O_4_ nanocrystals reacted for 10 and 20 h. **(d)** High-resolution TEM image. Inset, corresponding its fast Fourier transform indicating the particle is oriented along the zone axis [100].

Highly crystalline nanocrystals with a relatively narrow size distribution and reduced tendency toward aggregation were prepared for the purpose of generating a homogeneous 0–3 nanocomposite structure. Emphasis was on reducing the amount of surface passivation in the form of ligands, in order to optimize surface contact and therefore interaction with the ferroelectric polymer, following formation of the nanocomposite. The balance is in maintaining a highly disperse solvent suspension of the nanocrystals during combination with the polymer (which is aided by surface ligands) and obtaining a physical interaction between nanoparticle and polymer (hindered by long chain alkyl ligands and other typical reagents). Representative transmission electron micrograph (TEM, Figure 1a) illustrates that the samples consist of discrete, nanosized CoFe_2_O_4_ crystals with diameter of 8 to 18 nm. The particles are mostly spherical in shape and exhibit low size distribution. Following solvent evaporation, loose and localized aggregation occurs, possibly due to weak intermolecular interactions common and/or magnetic attraction amongst the nanoparticles. The chemical composition was obtained using energy-dispersive X-ray spectroscopy (EDX or EDS, Figure 1b): the ratio of the peaks is in good agreement with expected elemental composition. The average size determined by statistical analysis of the TEM images is consistent with that calculated by the Scherrer equation [18] from the XRD patterns (Figure 1c), indicating single crystallinity of the CFO nanoparticles. The position and relative intensity of all reflection peaks match well the cubic inverse spinel CoFe_2_O_4_ structure (PCPDS no. 04-006-4148), without indication of crystalline byproducts. The average diameter of the nanocrystals, calculated by the Scherrer formula [22] was determined to be 9 and 11 nm for 10- and 20-h treatments, respectively, indicating a slight increase in the average diameter with longer thermal treatment. The increase in particle dimension is ascribed to the longer reaction time, which allows and promotes the crystal growth after nucleation in the hydrothermal process. Images of isolated nanocrystals at higher magnification (HRTEM, Figure 1d) further confirm the crystallinity and phase purity of the as-synthesized cobalt ferrites. The well-defined two-dimensional lattice fringes of 10-nm nanocrystal indicate good crystallinity and lack of structural defects. The plane distance is measured as 2.99 Å, in good agreement with the (220) interplane spacing of the reported CoFe_2_O_4_ lattice.

**Figure 2:**
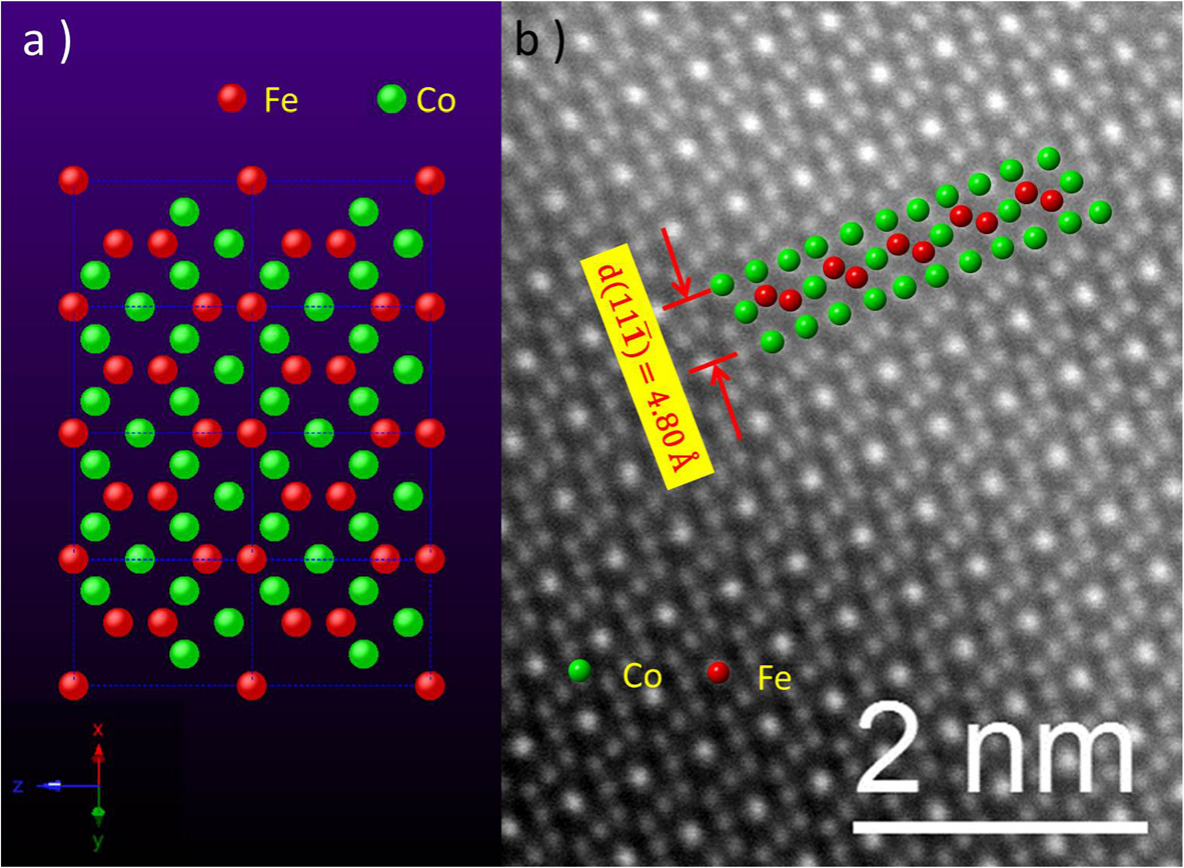
**Projection of the inverse spinel structure and the HAADF**-**STEM image of CoFe**_**2**_**O**_**4 **_**nanoparticles. (a)** Projection of the inverse spinel structure of CoFe_2_O_4_ along the <110> zone axis. Red balls represent iron atoms; green balls represent cobalt atoms; oxygen atoms have been removed for clarity. **(b)** Atomic resolution HAADF-STEM image of CoFe_2_O_4_ nanoparticles. Bright balls correspond to cobalt and ferrite atoms. Superimposed points are Co and Fe positions calculated from the crystal model.

Considering that the magnetic properties of the nanocrystal were to be compared that of the known bulk behavior of CoFe_2_O_4_, unequivocal identification of the crystal phase, symmetry, and composition of an individual nanocrystal was highly desirable. To further verify the crystal structure, the samples were studied by high angle annular dark field (HAADF) STEM and compared with a calculated model. Figure 2a illustrates the projection of the atomic structure model of CoFe_2_O_4_ along the <110 > zone axis, with oxygen atoms removed. Figure 2b shows the HAADF-STEM image of the as-synthesized nanocrystals, where the bright dots are Co and Fe atoms. The calculated positions of the transition metal atoms are superposed on the HAADF-STEM image, indicating that the elements and positions suggested in the model precisely fit those observed by STEM. As the intensity of the STEM pattern is proportional to *Z*^2^[23], where Z is the atomic number, O atoms are not visible, while Co and Fe atoms are present. Since the atomic numbers of Co (*Z* = 27) and Fe (*Z* = 26) are similar, it would be difficult to distinguish one from the other in the HAADF-STEM image. However, some Co columns exhibit stronger contrast than other Co/Fe columns in Figure 2b. This is because the former Co columns have twice the number of Co atoms as the dimmer ones. In addition, the measured interplane distance of (111) planes (4.80 Å) is consistent with the reported CoFe_2_O_4_ crystal information.

**Figure 3:**
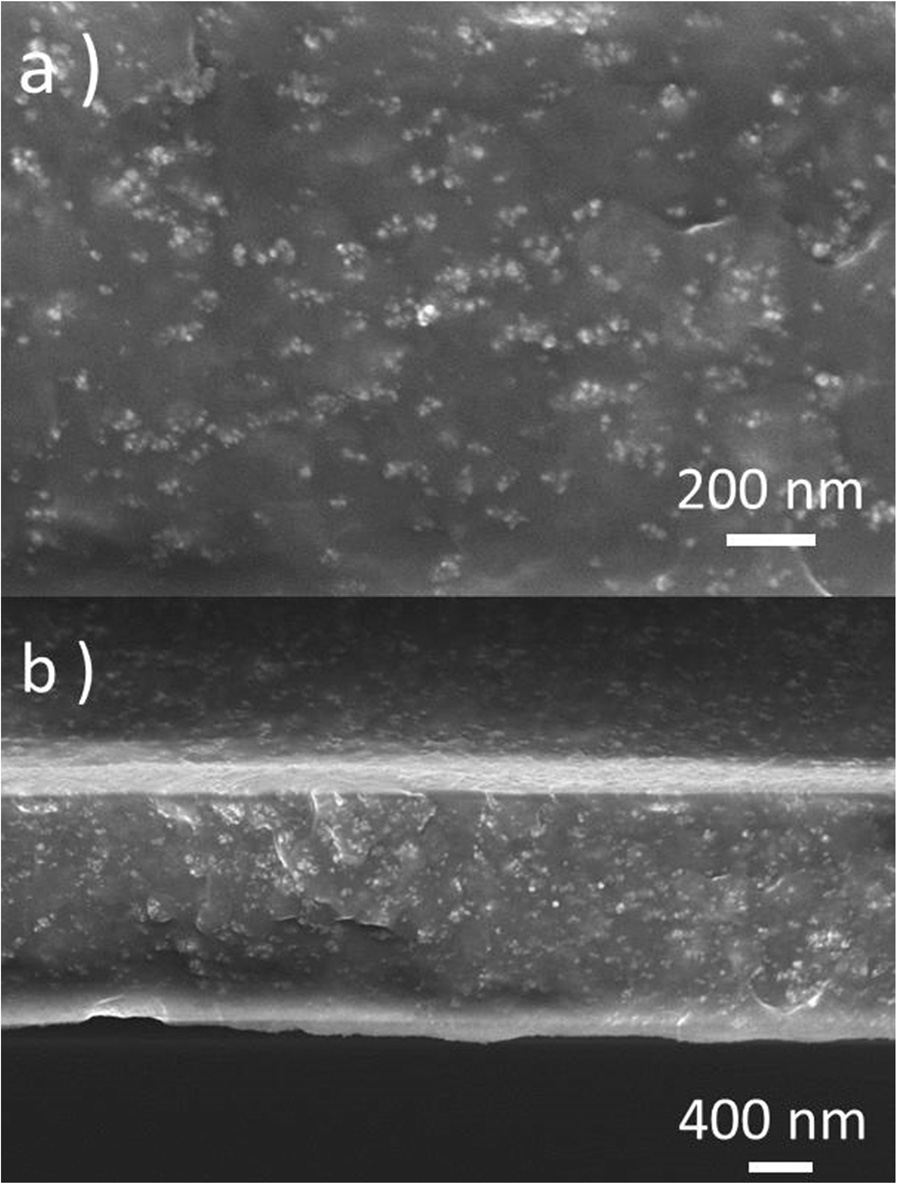
**SEM images of CoFe**_**2**_**O**_**4**_**/P(VDF**-**HFP) thin**-**films deposited on Si substrate.** With cobalt ferrite fraction of 25 wt.% and film thickness of 1.5 μm. **(a)** Top surface view; **(b)** cross-sectional view.

Continuous, uniform, and crack/void-free CoFe_2_O_4_/polymer films with thicknesses in the range 200 nm to 1.6 μm were systematically prepared by multiple spin/cast coating followed by thermal treatment to dry the film. Figure 3 shows SEM images with a CFO weight fraction of 25% where the white dots are the CFO nanoparticles and the dark background is the P(VDF-HFP) copolymer. The top surface view of the microstructure of the nanocomposite film demonstrates that monodisperse, ultrafine cobalt ferrite nanoparticles are well embedded in the polymer matrix, forming typical 0–3, particulate type nanocomposites. Loose agglomeration occurs locally due to the magnetic interaction among the nanopowders. Defects, pores, or phase separation unfavorable for device fabrication was not observed. The cross-sectional image (Figure 3b) confirms the thickness of the free standing film of approximately 1.5 μm. The observation of intimate physical contact between the CFO and P(VDF-HFP) phase components is a good starting point for attempting to generate mechanical, magnetic, or electrical coupling between them.

**Figure 4:**
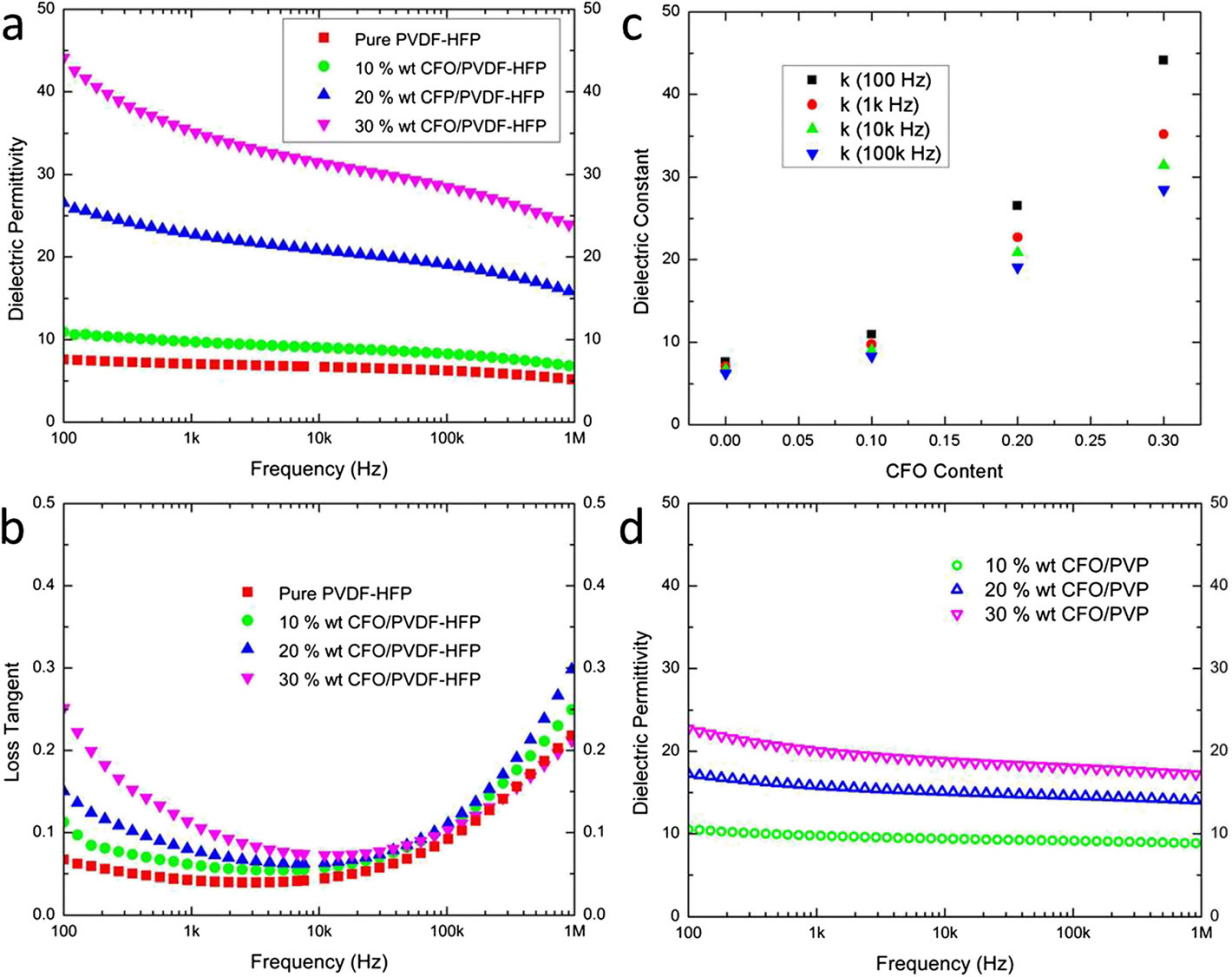
**Effective permittivity and loss tangent of the ferrites/polymer thin films.** Effective permittivity **(a)** and loss tangent **(b)** of CFO/P (VDF-HFP) nanocomposite thin films with CFO fractions from 0 to 30 wt.%. **(c)** Effective permittivity of the CFO/P(VDF-HFP) as a function of composition at 100 to 1 MHz. **(d)** Effective permittivity of CFO/PVP films.

The effective permittivity (*ϵ*_eff_) and loss tangent (tan *δ*) of the ferrites/polymer thin films (thickness of approximately 1 μm) were measured over the frequency range from 100 Hz to 1 MHz (Figure 4). Both the effective permittivity and loss tangent of the nanostructured films show a systemic increase as a function of the loading of CFO nanocrystals. The dielectric constant of the pure P(VDF-HFP) film is measured to be 8 at 100 Hz (Figure 4a), consistent with the reported data [24,25], and increases to 44 in the case of the 30 wt.% CFO samples due to the inclusion of the higher dielectric constant magnetic component (*k*(CoFe_2_O_4_) ≈ 400) [26]. The polarization in ferrites originates from the electronic exchange Fe^2+^ ⇔ Fe^3+^ and hole transfer between Co^2+^ ⇔ Co^3+^ in the spinel phase, which cannot follow the alternating external field beyond a certain frequency [27]. When the space charge carriers fail to keep up with the field and lag behind the alternation of its direction, the composites’ permittivity and loss tangent decrease monotonically with frequency. Once the frequency is over 10 kHz, the relaxation mechanism associated with the P(VDF-HFP) phase dominates the overall dielectric behavior [20]. The decrease in loss (Figure 4b) with frequency at low frequencies (<1 kHz) is attributed to the ionic DC conduction contribution from the P(VDF-HFP) copolymer phase, which yields interfacial or spatial charge polarization [28]. The increase in loss at high frequencies (>10 kHz) results from the *β* relaxation associated with the glass transition of the copolymer. Figure 4c further demonstrates the variation of dielectric constant as a function of CFO content in P(VDF-HFP). The nanocomposites show a low composition dependency at higher frequencies, since the dielectric behavior is dominated by the copolymer phase. The PVP films exhibit lower dielectric permittivity (Figure 4d) because the PVP polymer possesses a lower intrinsic dielectric constant of 5.1 (at 100 Hz) [29].

For 0–3 type nanocomposites with high permittivity nanocrystal fillers discretely distributed in a ferroelectric polymer matrix, the effective permittivity of the films is calculated by the modified Kerner model (or Kerner equation) [30,31] as shown in Equation 1:

(1) $$\epsilon_{\mathrm{eff}}=\frac{\epsilon_{h}f_{h}+\epsilon_{f}f_{f}\mathrm{AB}}{f_{h}+f_{f}\mathrm{AB}}$$

where

(2) $$A=\frac{3\epsilon_{h}}{\epsilon_{f}+2\epsilon_{h}}$$

and

(3) $$B=1+\frac{3f_{f}\left(\epsilon_{f}-\epsilon_{h}\right)}{\epsilon_{f}+2\epsilon_{h}}$$

The effective permittivity of the films, *ϵ*_eff_, is predicted using an average of the host and the filler particle permittivities (*ϵ*_h_ and *ϵ*_f_), wherein the contributions are weighted by the fraction of each component (*f*_f_ for filler and *f*_h_ for host, Equation 1). The measured effective permittivities and those calculated from the modified Kerner model for both PVDF-HFP and PVP films are summarized in Table 1.

**Table 1:** **Comparison of effective permittivity of the CFO**/**polymer films at 100 kHz from experimental and modified Kerner model**

**Sample**	***ϵ***_**eff **_**(measured)**	***ϵ***_**eff **_**(calculated from Kerner equation)**	**Δ*ϵ***_**eff**_
P(VDF-HFP) films			
10 wt.% CFO	9.1	7.3	+1.8
20 wt.% CFO	19.08	13.44	+5.64
30 wt.% CFO	28.56	19.71	+8.85
PVP films			
10 wt.% CFO	9.17	8.82	+0.35
20 wt.% CFO	14.59	13.62	+0.97
30 wt.% CFO	18.05	19.90	−1.85

The effective permittivity of the CFO/P(VDF-HFP) films shows a distinctive and continuous increase relative to the theoretical value estimated by the Kerner model, contrary to the expectations based solely on a composited effective dielectric constant. This can be contrasted with CFO/PVP, which shows significantly less deviation between experiment and theory, and follows expected behavior for a simple combination of two components for *ϵ*_*eff*_. This observation, of deviating behavior in the case of CFO/P(VDF-HFP), is interesting and strongly suggests additional interactions between the polymer and nanoparticle. The phenomenon is ascribed to interfacial interactions between the magnetic filler and the piezoelectric matrix. P(VDF-HFP) undergoes lattice distortion under an applied electric field due to the piezoelectric effect, which introduces local stresses and strain at the ferrite-copolymer interface. Since the thermal shrinkage nature of the P(VDF-HFP) makes complete mechanical coverage of the copolymer over the CFO nanocrystals, and both CFO and P(VDF-HFP) are mechanically hard phases, with Young’s modulus of 141.6 [32] and 237 GPa [33], respectively, the interfacial stress will be inversely applied to the copolymer phase via strong elastic interactions. As a result of the piezoelectric effect, the spatial charges and electric dipoles within the copolymer matrix are redistributed, manifested as variation of effective permittivities from the Kerner model. With higher ferrite contents, the interfacial elastic effect is stronger and leads to a more pronounced departure from the theoretical value.

**Figure 5:**
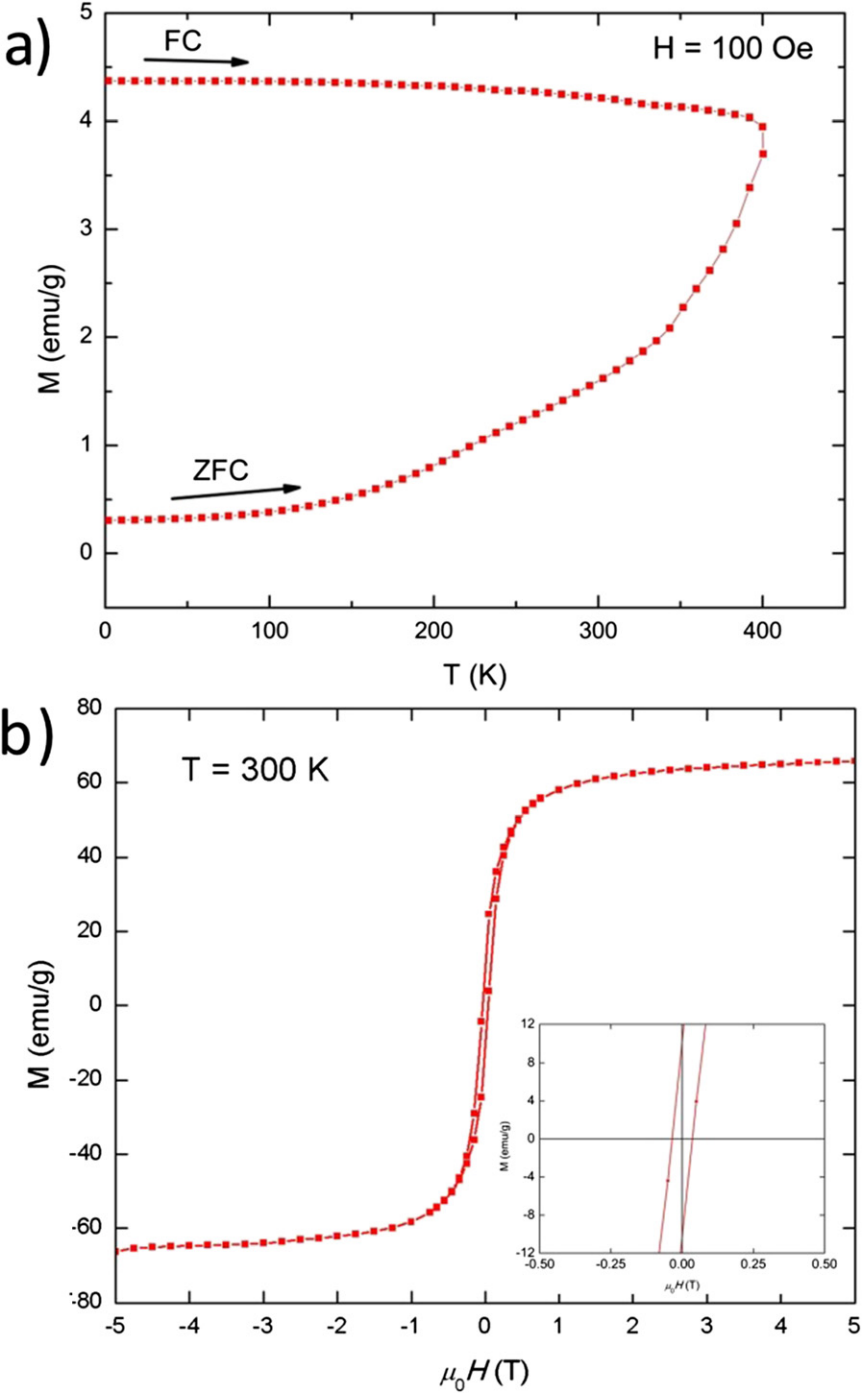
**Zero field**-**cooled and field**-**cooled (ZFC/FC) and room temperature magnetization curves (a) and hysteresis loop (b).** Measured for pure CoFe_2_O_4_ nanoparticles. Inset, central region on an expanded scale.

Magnetic measurements of the CoFe_2_O_4_ nanocrystals were conducted in both ZFC/FC, and hysteresis modes were analyzed. Figure 5a shows the low field (100 Oe) magnetization dependence with temperature (1.84 to 400 K) in ZFC/FC modes. After a ZFC process, the magnetization of the ferrite nanoparticles increases with rising temperature. Unlike other transition metal ferrite nanoparticles (e.g., Fe_3_O_4_[34], NiFe_2_O_4_[19], and MnFe_2_O_4_[35]), no maximum magnetization is detected in the ZFC process, indicating that the blocking temperature (*T*_b_) of CoFe_2_O_4_ nanoparticles is above 400 K, which is consistent with reported data of *T*_b_(CoFe_2_O_4_) = 525 K [19]. Additionally, an irreversible magnetic behavior is indicated by the splitting between the ZFC and FC curves. The irreversibility arises from the competition between the energy required for magnetic moment reorientation against the energy barrier associated with magnetoelectricity and the crystalline anisotropy. The field-dependent magnetization at ambient temperature (Figure 5b) shows a hysteresis with coercivity of 400 Oe, suggesting typical ferrimagnetic behavior. The coercivity represents the strength of the field that is needed to surpass the anisotropy barrier. The saturation magnetization (*M*_s_) and remnant magnetization (*M*_r_) is 66 and 10 emu/g, respectively, comparable with CoFe_2_O_4_ nanocrystals obtained by other approaches with similar sizes [15]. The *M*_s_ value of 66 emu/g is equivalent to magnetic moment dipole of 21.6 *μ*_B_ per cubic cobalt ferrite unit cell, which is 2.7 μ_B_ from each Co^2+^ ion. Generally Co^2+^ ions can offer three net spin magnetic moments. The lower value of magnetic moment and subsequent saturation magnetization of these CFO nanoparticles typically originates in the high surface area and concurrent surface disorder. At room temperature, the magnetic anisotropy prevents the magnetization direction of the nanocrystals to completely follow the direction of the external magnetic field.

**Figure 6:**
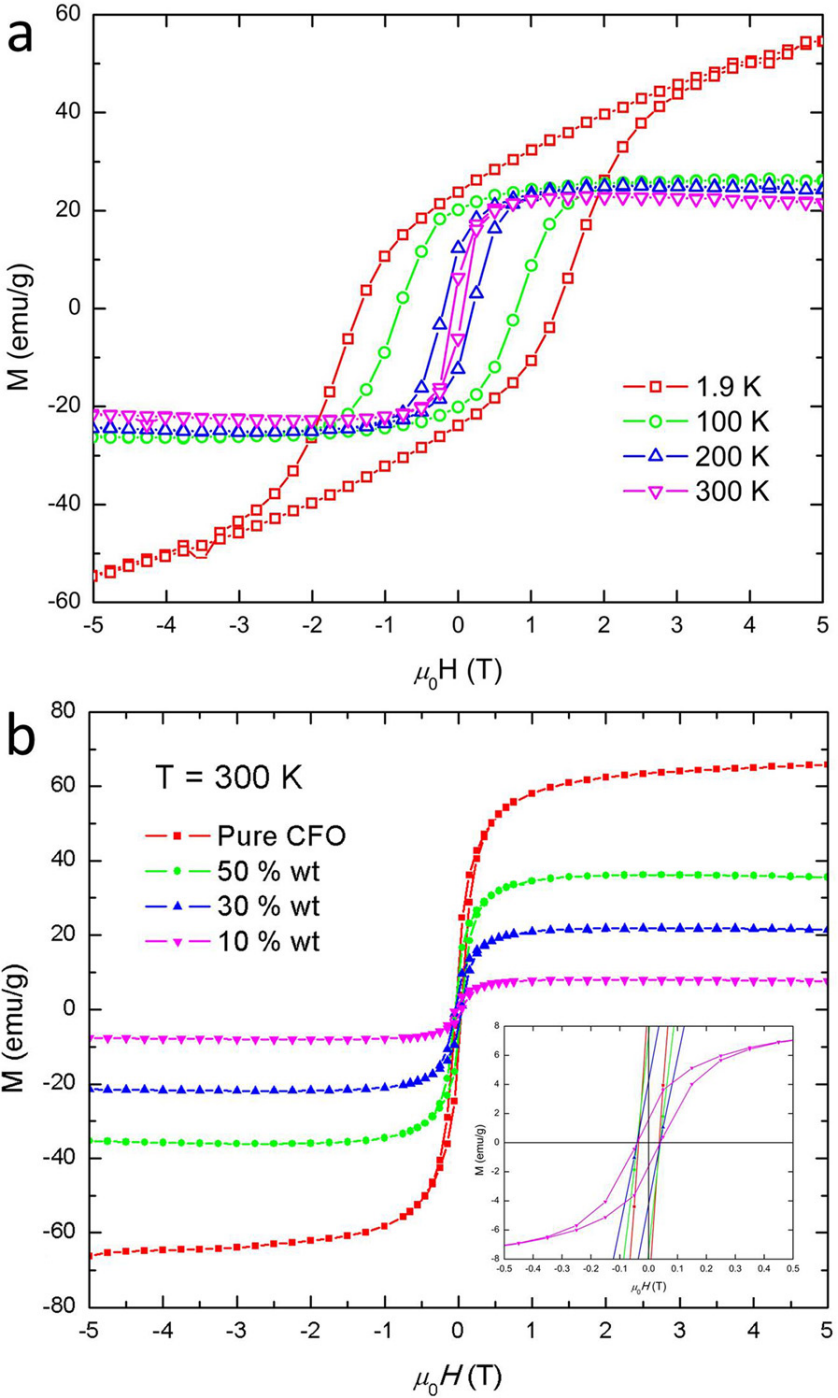
**Field**-**dependent magnetization hysteresis of CoFe**_**2**_**O**_**4**_**/P(VDF-HFP) nanocomposites. (a)** With 30 wt.% CFO loading at various temperatures and **(b)** at 300 K with various CFO weight fraction. Inset, central region on an expanded scale.

M(H) hysteresis loops of the CoFe_2_O_4_/P(VDF-HFP) and CFO/PVP nanocomposite thin films were recorded under an applied magnetic field up to 50 kOe. Figure 6a shows hysteresis loops of the 30 wt.% CoFe_2_O_4_/PVDF-HFP thin films at various temperatures, indicating typical ferri/ferromagnetic behavior. At 1.9 K, the 30 wt.% CFO/PVDF-HFP sample is not completely saturated at an applied magnetic field up to 50 kOe, while at higher temperatures (100 to 300 K), it gives a saturation magnetization (*M*_s_) of approximately 20 emu/g. The coercivity of the assembly is 400 Oe at 300 K and reaches 13 kOe at 1.9 K. Figure 6b shows the influence of the nanoparticle loading in the copolymer matrix to the saturation magnetization and remnant magnetization (*M*_r_). The increase in CFO phase content (as volume fraction) gives rise to a systematic increase in the overall M_s_ value; the non-magnetic P(VDF-HFP) polymer does not appear to inhibit the interactions of the magnetic polarization in individual nanocrystals. The composite films show the same coercivity, irrespective of the CFO content.

**Figure 7:**
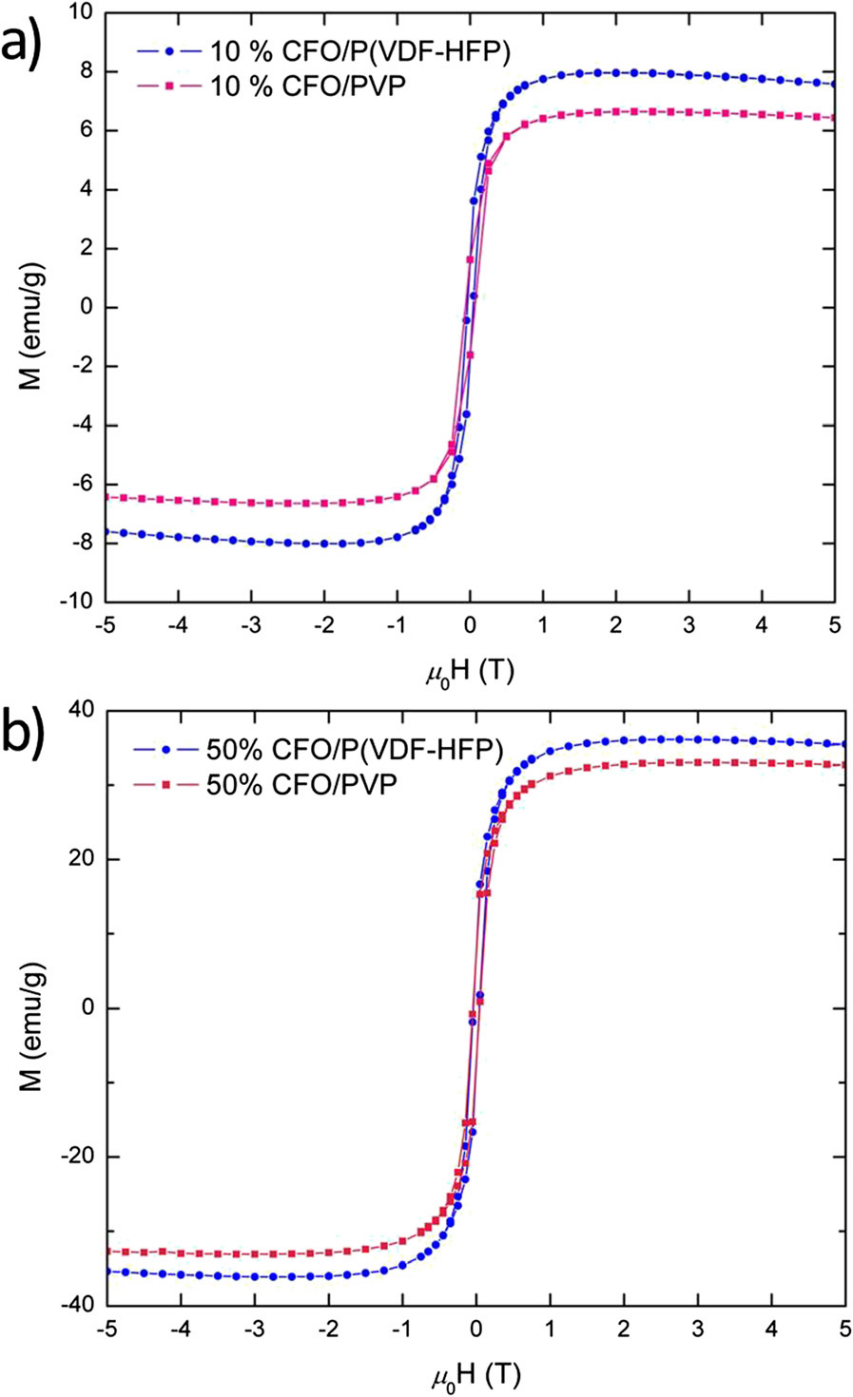
The hysteresis loops of 10 wt.% CFO/P(VDF-HFP) thin-films (a) and 50 wt.% CFO/PVP thin films (b).

In order to verify the concerted interaction between the magnetic and ferroelectric phases, hysteresis loops of the CFO/PVP nanocomposites were recorded (Figure 7) and compared with those of the CFO/P(VDF-HFP), presented in Table 2. The saturation magnetization of PVP films are lower compared to PVDF-HFP films with the same composition over the entire magnetic field range. The differences are +1.36 and +2.97 emu/g for 10 and 50 wt.% CFO loading, respectively. The change of the *M*_s_ values of the nanocomposite films was normalized for weight fraction and analyzed by the following equation:

**Table 2:** **Saturation magnetization** (***M***_**s**_) **and normalized percentage change of saturation magnetization** (**Δ*M***_**s**_%) **values for CFO**/**P**(**VDF**-**HFP**) **and CFO**/**PVP films with various CFO contents**

**Sample**	***M***_**s **_**(emu/g)**	**Δ*M***_**s**_**%**
P(VDF-HFP) films		
10 wt.% CFO	8.0	+20.7%
30 wt.% CFO	21.8	+9.61%
50 wt.% CFO	36.0	+8.60%
PVP films		
10 wt.% CFO	6.6	+0.09%
30 wt.% CFO	20.2	+0.96%
50 wt.% CFO	33.0	−0.36%

(4) $$\Delta M_{s}\%=\frac{\frac{M_{s}}{f}-M_{s0}}{M_{s0}}$$

where *M*_s_ is the saturation magnetization of a film with certain CFO weight fraction, *f* is the corresponding weight percentage, *M*_s0_ is the saturation magnetization of pure CFO, and Δ*M*_s_% is the normalized percentage change of the *M*_s_ value of each polymer-based film relative to the comparative weighted, pure cobalt ferrite films. The Δ*M*_s_% values for both P(VDF-HFP) and PVP films are summarized in Table 2. The Δ*M*_s_% for the CFO/PVP films is close to zero for all three samples, indicating that the net magnetic moments of the thin films is equivalent to the sum of the contributions from each individual CFO grain inside the PVP matrix (volume fraction contribution only). In contrast, all CFO/P(VDF-HFP) films exhibit positive values of Δ*M*_s_%, with a gradual increase as the copolymer fraction increases. This observation was carefully calibrated against possible errors in the calculation of the volume fraction, most notably through measurement of the film thickness by SEM (see ‘Methods’ section). This observation strongly suggests a contribution to the Δ*M*_s_ due to the presence of P(VDF-HFP) in the form of an enhancement of the saturation magnetization of the composite, with the enhancement stronger for samples with a lower CFO:P(VDF-HFP) ratio.

CoFe_2_O_4_ nanocrystal powders show less than 1% variation in hysteresis loops, whereas CFO/P(VDF-HFP) films show enhancements up to 20.7% in Δ*M*_s_. The enhancement of the *M*_s_ value from the P(VDF-HFP) phase, we believe, is a concerted effect and is evident of a ME effect, specifically, through inverse magnetorestrictive coupling. First, the magnetostrictive effect induces a distortion of the crystal lattices of CoFe_2_O_4_ under an applied magnetic field, which in turn leads to local strains or stresses of between the piezoelectric and magnetic phases via intimate mechanical contact. The hypothesis of the influence of intimate mechanical contact between nanocrystals and P(VDF-HFP) is already supported by the observation of permittivity changes unexplained by volume fraction alone, described above. We postulate that the interfacial stress is inversely applied on the CFO phase, which further leads to the change of domain magnetization as a result of an inverse magnetostrictive effect. The effect is quantified by Equation 5:

(5) $$E=\frac{3}{2}\lambda_{s}\;\sigma {\;\sin}^{2}\theta$$

where *E* is the magnetic strain energy density, *λ*_s_ is the magnetostrictive expansion at saturation, *θ* is the angle between the saturation magnetization, and *σ* is the stress applied on a single magnetic domain [36]. With limited expansion allowed by intimate contact of two hard phases, when compression is applied to CFO phase, the energy is minimized when magnetization is parallel to *σ* (*θ* = 0). Consequently, *M*_s_ is increased by tension. Moreover, in a sample of pure CFO nanoparticles (*M*_s_ = 66 emu/g) each Co^2+^ ion exhibits a magnetic moment of 2.7 μ_B_, while in the 10 wt.% CFO/P(VDF-HFP)) films (*M*_s_ = 8.0 emu/g), the Co^2+^ ion shows a net magnetic moment of 3 μ_B_, which equals the maximum magnetic moment a Co^2+^ ion can offer in the inverse spinel structure. This observation indicates that by intimate mechanical coverage of the CFO particles, P(VDF-HFP) reduces the nanocrystals’ degree of surface disorder and surface anisotropy via redistributing charges and dipoles within the copolymer matrix, which allows the magnetization of the cobalt ions to completely follow the external magnetic field. Additionally, as the content of cobalt ferrite nanoparticles increases, the particles’ tendency towards agglomeration increases. The interfacial area is reduced due to the formation of small clusters of nanoparticles, and therefore, the interfacial interaction is weakened. This explains why the *M*_s_ enhancement is strongest in the 10 wt.% sample (+20.7%), in which the nanoparticles are more completely dispersed, compared to 30 and 50 wt.% samples (+9.6% and +8.6%, respectively).

The magnetoelectric effect associated with the magnetostrictive/piezoelectric coupling typically can only be observed under high magnetic fields at a very low temperature. In this work, the nanocomposite thin films show substantial magnetoelectric coupling at room temperature. The piezoelectric properties of P(VDF-HFP) and ferrimagnetic properties of CoFe_2_O_4_ nanocrystals are ideal and complimentary in this respect, resulting an observable magnetoelectric coupling.

## Conclusions

Crystalline ultrafine CFO with a relatively narrow size distribution from 8 to 18 nm were dispersed in a P(VDF-HFP) copolymer host, forming 0–3 particulate type magnetoelectric nanocomposite thin films. The resulting films exhibit composition-dependent effective permittivity and loss. Following full structural characterization, the magnetic properties of the pure CoFe_2_O_4_ nanoparticles were studied and it was confirmed that the saturation magnetization and ZFC/FC curves demonstrate typical ferrimagnetic behavior. By comparing the P(VDF-HFP) and PVP samples, a clear difference in the behavior of the nanocomposite films with respect to effective permittivity and saturation magnetization is observed, highlighting the difference between the use of the ferroelectric polymer and the non-ferroelectric polymer. A magnetoelectric coupling is believed to be observed in the case of CFO/P(VDF-HFP). The origin of the magnetoelectric coupling is attributed to strong elastic interactions between the electric and magnetic phases. The nanocomposite, given its room temperature properties, is an interesting candidate magnetoelectric material with applications in smart devices such as sensors.

## Abbreviations

CFO: CoFe_2_O_4_; EDX: Energy-dispersive X-ray spectroscopy; HAADF-STEM: High angle annular dark field scanning transmission electron microscopy; HRTEM: High-resolution transmission electron microscopy; SEM: Scanning electron microscopy; STEM: Scanning transmission electron microscopy; TEM: Transmission electron microscopy; XRD: X-ray diffraction.

## Competing interests

The authors declare that they have no competing interests.

## Authors’ contributions

XL carried out nanoparticle synthesis, thin film fabrication, dielectric properties, and measurements and drafted the manuscript. SL participated in dielectric/magnetic properties characterization and discussion and idea/experiment design. MGH carried out HRTEM and HAADF-STEM analysis, with XL assisting. LZ and HD carried out the magnetic property tests, with XL assisting. JL, YZ, and LKE helped to supervise the experiments and participated in the design of the study and manuscript revision. SO conceived of the study, supervised the project and experiments, and helped to write the manuscript. All authors read and approved the final manuscript.
